# A comparative study of anaerobic fixed film baffled reactor and up-flow anaerobic fixed film fixed bed reactor for biological removal of diethyl phthalate from wastewater: a performance, kinetic, biogas, and metabolic pathway study

**DOI:** 10.1186/s13068-017-0826-9

**Published:** 2017-05-31

**Authors:** Samira Yousefzadeh, Ehsan Ahmadi, Mitra Gholami, Hamid Reza Ghaffari, Ali Azari, Mohsen Ansari, Mohammad Miri, Kiomars Sharafi, Soheila Rezaei

**Affiliations:** 1 Department of Environmental Health Engineering, Aradan School of Public Health and Paramedical, Semnan University of Medical Sciences, Semnan, Iran; 2 Department of Environmental Health Engineering, School of Public Health, Tehran University of Medical Sciences, Tehran, Iran; 3 Students’ Scientific Research Center (SSRC), Tehran University of Medical Sciences, Tehran, Iran; 4 Department of Environmental Health Engineering, School of Public Health, Kashan University of Medical Sciences, Kashan, Iran; 5 Occupational Health Research Center (OHRC), Iran University of Medical Sciences, Tehran, Iran; 6 Department of Environmental Health Engineering, School of Public Health, Iran University of Medical Sciences, Tehran, Iran; 7 Department of Environmental Health Engineering, Faculty of Health, Hormozgan University of Medical Sciences, Bandar Abbas, Iran; 8 Environmental Science and Technology Research Center, Department of Environmental Health, School of Public Health, Shahid Sadoughi University of Medical Sciences, Yazd, Iran; 9 Department of Environmental Health, School of Public Health, Sabzevar University of Medical Sciences, Sabzevar, Iran; 10 Research Center for Environmental Determinants of Health (RCEDH), Kermanshah University of Medical Sciences, Kermanshah, Iran; 11 Social Determinants of Health Research Center, Yasuj University of Medical Sciences, Yasuj, Iran

**Keywords:** Biofilm, Diethyl phthalate, Wastewater, Anaerobic treatment, Organic loading rate, Hydraulic loading rate, Biodegradation, Phthalic acid esters

## Abstract

**Background:**

Phthalic acid esters, including diethyl phthalate (DEP), which are considered as top-priority and hazardous pollutants, have received significant attention over the last decades. It is vital for industries to select the best treatment technology, especially when the DEP concentration in wastewater is high. Meanwhile, anaerobic biofilm-based reactors are considered as a promising option. Therefore, in the present study, for the biological removal of DEP from synthetic wastewater, two different anaerobic biofilm-based reactors, including anaerobic fixed film baffled reactor (AnFFBR) and up-flow anaerobic fixed film fixed bed reactor (UAnFFFBR), were compared from kinetic and performance standpoints. As in the previous studies, only the kinetic coefficients have been calculated and the relationship between kinetic coefficients and their interpretation has not been evaluated, the other aim of the present study was to fill this research gap.

**Results:**

In optimum conditions, 90.31 and 86.91% of COD as well as 91.11 and 88.72% of DEP removal were achieved for the AnFFBR and UAnFFFBR, respectively. According to kinetic coefficients (except biomass yield), the AnFFBR had better performance as it provided a more favorable condition for microbial growth. The Grau model was selected as the best mathematical model for designing and predicting the bioreactors’ performance due to its high coefficients of determination (0.97 < *R* ^2^). With regard to the insignificant variations of the calculated Grau kinetic coefficients (*K*_G_) when the organic loading rate (with constant HRT) increased, it can be concluded that both of the bioreactors can tolerate high organic loading rate and their performance is not affected by the applied DEP concentrations.

**Conclusions:**

Both the bioreactors were capable of treating low-to-high strength DEP wastewater; however, according to the experimental results and obtained kinetic coefficients, the AnFFBR indicated higher performance. Although the AnFFBR can be considered as a safer treatment option than the UAnFFFBR due to its lower DEP concentrations in sludge, the UAnFFFBR had lower VSS/TSS ratio and sludge yield, which could make it more practical for digestion. Finally, both the bioreactors showed considerable methane yield; however, compared to the UAnFFFBR, the AnFFBR had more potential for bioenergy production. Although both the selected bioreactors achieved nearly 90% of DEP removal, they can only be considered as pre-treatment methods according to the standard regulations and should be coupled with further technology.

**Electronic supplementary material:**

The online version of this article (doi:10.1186/s13068-017-0826-9) contains supplementary material, which is available to authorized users.

## Background

Environmental and water pollution has become an issue of serious international concern in recent years [1–5]. In this regard, legislation requirements for discharging wastewater have recently become much stricter [6].

Emerging synthetic chemicals have entered into the environment through industrial activities [6, 7]. One of these emerging synthetic compounds is phthalic acid esters (PAEs), commonly named phthalates [8], which are widely used as plasticizers [9, 10] and globally applied in large quantities to make products such as plastics, pesticides, adhesives, paints, and cosmetics [11–13]. PAEs are ubiquitous pollutants as they have extensive applications in industrial processes, are not chemically bonded to products, and can migrate to environments [6, 14].

In recent years, PAEs have been considered as hazardous environmental pollutants [8]. They can have carcinogenic, mutagenic, and teratogenic potential impacts due to their ability to disrupt endocrine function [15–17]. Moreover, they can cause adverse health problems in humans’ reproductive and developmental systems [18–21] and have been associated with type 2 diabetes [22].

Therefore, some regulatory organizations including China National Environmental Monitoring Center (CNEMC), the United State Environment Protection Agency (USEPA), and the European Union have classified PAEs as top-priority pollutants [23].

Diethyl phthalate (DEP) is one of the important phthalates, the concentration of which was reported to be about 500 mg L^−1^ in discharged industrial effluent [8].

Although phthalates may not be significantly eliminated by physico-chemical processes like hydrolysis and photolysis, and other advanced treatment processes such as membrane and oxidation techniques are not advisable due to their high investment and operation costs, it has been shown that microbial biodegradation can play an important role in removing these pollutants from various environments [8, 24–26].

Meanwhile, a safe and cost-effective biological wastewater treatment is an important issue to consider [27, 28], especially for the industries which have to find financially feasible treatment methods to meet the permissible discharge levels [29]. Anaerobic biodegradation of organic matters in wastewater is an economic way for wastewater remediation [30].

The logical reasons to use anaerobic treatment processes can be explained by considering the advantages of these processes which are related to their low energy demand, less sludge production, bioenergy (methane and hydrogen) production, and low nutrient requirement [31–36]. Furthermore, industrial wastewaters with high chemical oxygen demand (COD) containing high concentration of pollutants cannot be easily biotreated by aerobic bioreactors; this is while anaerobic bioreactors have been preferably and successfully applied to such wastewater [30, 31] and organic compounds of the mentioned wastewaters are ideal for green bioenergy production [37].

Attached growth (biofilm) anaerobic bioremediation has additional advantages over suspended growth bioreactors, including more stability in operation, higher tolerance for pH, temperature, and toxic shocks, higher utilization rate of substrate, and ability to retain high biomass concentration under shorter hydraulic retention time (HRT) and overcome wash-out issues [30, 31, 37–39].

As highlighted above, microbial biodegradation can play an important role in removing PAEs and it is vital for industries to select the best treatment technology for all the wastewater with high concentration of phthalates [6]. Therefore, the main aim of the present study was to evaluate the performance of two different anaerobic biofilm bioreactors including anaerobic fixed film baffled reactor (AnFFBR) and up-flow anaerobic fixed film fixed bed reactor (UAnFFFBR), which have promising performance and have been recently used for industrial wastewater [40–42]. Moreover, as kinetic coefficients and mathematical modeling are two crucial tools that can be used to predict the performance of bioreactors and optimize their design [43, 44], they were used for comparing the selected bioreactors, since they can represent the microbial activity in different environmental conditions provided by the selected bioreactors. As kinetic and mathematical modeling directly relies on removal efficiency and microbial mass, they preferably, practically, and easily can be used by the designers and operators of treatment plants, while the recognition of microbial type and their number cannot be used for predicting the performance of bioreactors [30, 45]. In addition, there are many analytical and conceptual challenges for profiling the diversity of microbial communities and diagnosis of their species in complex microbial communities such as wastewater [46, 47].

While many studies have been conducted to find a practicable way for evaluating the behavior of bioreactors applied to wastewater bioremediation, they could not completely evaluate the bioreactors’ performance in terms of kinetic coefficients and mathematical modeling. Moreover, other studies have been only focused on calculating critical kinetic coefficients and computing removal efficiency, and have not investigated and evaluated their variations [48–50]. In addition, selecting the best bioreactors is traditionally done with their performance like COD removal, while their kinetic coefficients and bioenergy production are not usually considered very well. It should be noted that we previously applied mathematical modeling and kinetic evaluation for comparing the biodegradability of different PAEs as substrates [51]; to our best knowledge, it is the first study for comparing the performance of the selected bioreactors by the mentioned methods. It should also be noted that there are other important parameters like the produced sludge quality and bioenergy production which need to be considered. Therefore, to fill the mentioned research gaps, this study was mainly aimed to compare two biofilm-based reactors including anaerobic fixed film baffled reactor (AnFFBR) and anaerobic fixed film fixed bed reactor (UAnFFFBR) for DEP removal from synthetic wastewater.

## Methods

### Implementation and operation of bioreactors

Two laboratory-scaled rectangular-shape bioreactors with the identical 6 L operating volume were used in this study. The reactors were made of 4 mm-thick Plexiglas. The AnFFBR was divided into three equal compartments by vertical baffles (each part contained 2 L operating volume and split by a further baffle) which were connected internally.

The dimensions of UAnFFFBR were 10 (length) × 10 (width) × 70 cm (height) (Additional file 1: Figure S1).

Both bioreactors were seeded with an anaerobic sludge obtained from a full-scale municipal wastewater treatment plant (Ekbatan, Iran). Synthetic wastewater was continuously pumped from the feed tank into the AnFFBR using a dosing pump (Etatron, Italy). Another identical dosing pump was also used to feed the UAnFFFBR. For inoculating both the bioreactors (start-up phase), further dosing pumps were used to recycle the washed-out sludge from the settling tank.

The bioreactors were operated at 25 ± 2 °C and fitted with the heaters to maintain the reactors’ temperature stable. The bioreactors were filled by high-density polyethylene (HDPE) carriers, which acted as a fixed bed for biofilm (microbial) growth. The carriers had approximately 0.95–0.98 g cm^−3^ and 535 m^2^ m^−3^ of density and active surface area, respectively. The bioreactors were filled by 1.6 m^2^ of the carriers’ available surface area (50% filling ratio).

In the acclimation step, glucose corresponding to 600 mg L^−1^ of chemical oxygen demand (COD) was used as the primary substrate and sole carbon source. Then, after reaching the steady-state condition, DEP was added stepwise and glucose concentration was reduced in parallel until DEP formed the entire carbon source. The experiments were conducted in two different study steps. The effects of HRT and organic loading rate (different concentrations of DEP) on the performance of the selected bioreactors were evaluated in study steps (A) and (B), respectively.

To have the chemical oxygen demand/nitrogen/phosphorous ratio of 350/5/1, ammonium chloride (NH_4_Cl) and ammonium bicarbonate (NH_4_HCO_3_) (for nitrogen source) along with mono-potassium phosphate (KH_2_PO_4_) (for phosphorous source) were used as nutrients in all the study steps. The composition of trace elements in synthetic wastewater was selected as follows: CaCl_2_·2H_2_O (14 mg), MgSO_4_·7H_2_O (90 mg), and 0.3 mL of trace solution per liter of synthetic wastewater. The following compounds were dissolved per liter to prepare the trace solution: KI (0.18 g), MnCl_2_·H_2_O (0.12 g), FeCl_3_·6H_2_O (1.5 g), CuSO_4_·5H_2_O (0.03 g), H_3_BO_3_ (0.15 g), CoCl_2_·6H_2_O (0.15 g), ZnSO_4_·7H_2_O (0.12 g), Na_2_MoO_4_·2H_2_O (0.06 g), and EDTA (10 g) [52]. Furthermore, NaHCO_3_ was applied to adjust pH at 7.5 ± 0.2.

### Kinetics and mathematical modeling

Critical kinetic parameters and mathematical models have important impacts for predicting and designing biological wastewater treatment plants.

In the present study, three common substrate removal models, namely Stover-Kincannon, first order, and Grau (second order), were used to design the bioreactors and predict substrate removal rate. Under steady-state conditions ($\frac{{{\text{d}}S}}{{{\text{d}}t}} = 0$), if the first-order model prevails, the substrate consumption rate can be predicted by Eq. (1). Furthermore, Eq. (2) is the simplified form of Eq. (1):

1 $$- \frac{{{\text{d}}s}}{{{\text{d}}t}} = \frac{{Q \cdot S_{0} }}{V} - \frac{Q \cdot S}{V} - K_{1} S$$

2 $$\frac{{S_{0} - S}}{\text{HRT}} = K_{1} S,$$

where *K*_1_ is the kinetic constant for the first-order model (day^−1^), *S*_0_ and *S* are the influent and effluent substrate concentrations (mg L^−1^), respectively, *Q* is the inflow rate (L day^−1^), *V* is the reactor volume (liter), and HRT is the hydraulic retention time (day).

In the modified Stover–Kincannon model, the substrate utilization rate for the biofilm-based bioreactors can be determined by Eq. (3) which is based on organic loading rate. Equation (4) is also the linearized form of Eq. (3):

3 $$\frac{{{\text{d}}S}}{{{\text{d}}t}} = \frac{{U_{\text{max} } \times \left( {Q \times \frac{{S_{0} }}{V}} \right)}}{{K{}_{\text{B}} + \left( {Q \times \frac{{S_{0} }}{V}} \right)}}$$

4 $$\frac{V}{{Q \times (S_{0} - S)}} = \frac{{K_{\text{B}} }}{{U_{ \text{max} } }} \times \frac{V}{{Q \times S_{0} }} + \frac{1}{{U_{\text{max} } }}$$

where *U*_max_ is the maximum removal rate of the substrate (mg COD L^−1^ day^−1^) and *K*_B_ is the saturation value constant (mg L^−1^ day^−1^). After the linear regression of Eq. (4), these coefficients can be calculated from the intercept and slope of the linear graph, respectively.

Equations (5) and (6) represent the second-order substrate removal model proposed by Grau. Equation (6) is also the integrated form of Eq. (5):

5 $$\frac{{ - {\text{d}}S}}{{{\text{d}}t}} = K_{\text{G}} \times X \times \left( {\frac{S}{{S_{0} }}} \right)^{2}$$

6 $$\frac{\text{HRT}}{E} = \left( {n \times {\text{HRT}}} \right) + m$$

where *n* (dimensionless) and *m* (day^−1^) are the constants for a second-order model which can be determined from the intercept and slope of the plotted line of HRT versus $\frac{{(S_{0} \times {\text{HRT}})}}{{(S_{0} - S)}}$. Moreover, (*X*) is the suspended biomass concentration (mg VSS L^−1^), (*E*) is the fractional substrate removal efficiency (dimensionless), and *K*_G_ is the Grau second-order substrate removal rate constant (day^−1^). *K*_G_ can be obtained from the following equation [53]:

7 $$m = \frac{{S_{0} }}{{K_{\text{G}} \times X}}.$$

Other kinetic coefficients including (*K*_S_) and (*K*) in the biofilm-based reactors can be obtained by combining mass balance equation [Eq. (8)] with Monod equation [Eq. (9)]. *r*_s_ is the substrate utilization rate (g m^−2^ day^−1^).

8 $$V\frac{{{\text{d}}S}}{{{\text{d}}t}} = QS_{0} - QS - A(r_{\text{s}} )$$

9 $$r_{\text{s}} = - \frac{{{\text{d}}s}}{{{\text{d}}t}} = \frac{{K \cdot S \cdot X_{\text{att}} }}{{K_{\text{s}} + s}}.$$

In steady-state conditions, the substrate concentration changes in Eq. (8) can be ignored $(\frac{{{\text{d}}S}}{{{\text{d}}t}} = 0)$ and Eqs. (8) and (9) given above can be combined as the following equation:

10 $$\frac{1}{S} = \frac{k}{{K_{\text{s}} }}\left( {\frac{{A \cdot X_{{(_{A} )}} }}{{Q(S_{0} - S)}}} \right) - \frac{1}{{K_{\text{s}} }}.$$

Finally, *K*_S_ as the half saturation constant (mg L^−1^) and (*K*) as the overall reaction rate (day^−1^) can be calculated from the linear regression of plotting $\frac{1}{S}$ versus $\frac{{X_{\text{att}} }}{{Q(S_{0} - S)}}$ line. Furthermore, *X*_att_ which is the attached mass of biofilm (g VS) can be calculated by multiplying *A* (total available area, m^−2^) by *X*_*A*_:

11 $$X_{\text{att}} = X_{A} \times A.$$

Moreover, (*Y*) and (*K*_d_) as the biomass yield coefficient (g VS produced/g substrate consumed) and biomass decay rate (day^−1^), respectively, can be calculated by mass balance equation (Eq. 12) and Monod growth kinetic (Eq. 13) [8]:

12 $$V\frac{{{\text{d}}X}}{{{\text{d}}t}} = QX_{0} - QX_{\text{e}} + A(r_{\text{g}} )$$

13 $$r_{\text{g}} = Y\left( {r_{\text{su}} } \right) - K_{\text{d}} \cdot A \cdot X_{(A)}$$

where *r*_g_ and *X*_e_ are the specific growth rate (g VSS m^−2^ day^−1^) and sloughed VS from the reactor (g VS day^−1^). If steady-state conditions are achieved ($\frac{{{\text{d}}X}}{{{\text{d}}t}}$ = 0), Eq. (14) can be obtained by combining Eqs. (12) and (13) as follows:

14 $$\frac{{(S_{0} - S)}}{{X_{\text{e}} }} = \frac{{K_{\text{d}} }}{Y}\left( {\frac{{X_{\text{att}} }}{{Q \cdot X_{\text{e}} }}} \right) + \frac{1}{Y}.$$

Finally, by the linear regression of Eq. (14), (*Y*) and (*K*_d_) can be determined.

Subsequently, (*µ*_m_) coefficient which is the maximum specific growth rate (maximum specific growth rate, day^−1^) can be calculated by multiplying (*K*) and (*Y*) coefficients expressed in Eq. (15). Eventually, influent substrate utilization rate can be calculated from Eq. (16), obtained by combining Eq. (9) with Eq. (14) [30, 49]:

15 $$\mu_{\text{m}} = K \cdot Y$$

16 $$r_{\text{su}} = \frac{{\mu_{\text{m}} \cdot X \cdot S_{0} }}{{Y(K_{\text{s}} + S_{0} )}}.$$

### Analytical methods

COD and biofilm characteristics including total solids (TS) and volatile solids (VS) were analyzed by the analytical techniques of standard methods [54].

Total organic carbon (TOC) samples were filtered by a 0.45 µm filter and, subsequently, analyzed by TOC-Vcsh (Shimadzu, Japan) to measure the extend of mineralization.

Biogas measurements were performed as described in the study of Lay et al. [55].

Moreover, the analytical measurement of diethyl phthalate was performed using a gas chromatograph (GC) which was coupled with a flame ionization detector (FID) and equipped with a HP-5 capillary column.

To measure the DEP concentration, 10 mL of the wastewater sample was filtered through a glass fiber filter with 0.7 µm pore size and, subsequently, extracted by 2 mL of *n*-hexane solution. Finally, 2 µL of the extracted sample was injected into GC-FID. Concentration of the samples was measured by comparing them to the standard curve prepared at five points. Naphthalene was added as the internal standard.

GC temperature program was as follows: the oven’s initial temperature was set at 70 °C for 1 min and, then, followed by a 10 °C min^−1^ ramp to 250 °C as its final temperature and was maintained at this temperature for 2 min. The injector and detector temperatures were set at 250 and 260 °C, respectively. To quantify the DEP concentration in effluent sludge (sloughed biofilm), 1 g of the freeze-dried sludge sample was ground and, subsequently, extracted by 10 mL of *n*-hexane.

To detect the metabolites of biodegradation, a gas chromatograph system equipped with a mass spectrometer (MS) detector and liquid chromatography tandem–mass spectrometry (LC–MS/MS) were used.

## Results and discussion

### Evaluating performance of bioreactors

Experimental results for the AnFFBR and UAnFFFBR performance in terms of removing diethyl phthalate under different operation conditions including different HRTs and influent DEP concentrations are shown in Tables 1 and 2, respectively. It should be noted that data presented in Table 2 [study step (A)] which obtained under different HRTs have previously been used for comparing biodegradability of different PAEs [51]. The concentrations of COD, TOC, and DEP in the effluent of the bioreactors were determined as the bioreactor responses. The initial analysis for the COD and TOC experiments showed that each 1 g DEP L^−1^ produced 1.85 g COD L^−1^ and 0.62 g TOC L^−1^, respectively.

**Table 1:** Performance of anaerobic fixed film baffled reactor (AnFFBR) in removing DEP

Study step	A					B			
Study phase	1	2	3	4	5	1	2	3	4
Influent DEP concentration (mg L^−1^)	300	300	300	300	300	400	500	600	700
HRT (h)	12	18	24	30	36	36	36	36	36
*L*_org_ (g COD m^−2^ day^−1^)^a^	4.162	2.775	2.081	1.665	1.387	1.85	2.312	2.775	3.237
Attached mass [TS (mg) of biofilm]	6180	4860	4180	3880	3710	4560	5400	6260	7170
Attached mass [VS (mg) of biofilm]	4500	3300	2820	2520	2400	3120	3900	4560	5340
VS/TS ratio	0.728	0.679	0.674	0.649	0.647	0.684	0.722	0.728	0.744
Effluent total suspended solids (mg day^−1^)	334.3	205	137.7	111.4	90	141.6	168.8	239.5	276.2
Effluent volatile suspended solids (mg day^−1^)	224.4	130.2	84.5	64.2	48.4	81	98.6	144.3	168.9
DEP concentration in TSS_e_ (mg g^−1^)^b^	8.5	8.2	6.6	6.2	4.1	7.3	7.5	7.5	7.9
SRT (day)	18.48	23.7	30.35	34.83	41.22	32.2	31.99	26.13	25.96
Methane production (L/g COD_rem_)	0.21 (0.19)^c^	0.28 (0.25)	0.32 (0.29)	0.37 (0.34)	0.44 (0.4)	0.38 (0.35)	0.35 (0.32)	0.34 (0.31)	0.31 (0.28)
Methane percentage (%)	42.2	44.2	52.5	56.4	64.8	63.3	62.1	61.7	61.1
DEP removal (%)	73.46	78.43	81.4	86.33	90.26	90.62	90.64	90.83	91.11
COD removal (%)	66.66	70.1	77.33	82.29	87.01	88.04	89.48	89.6	90.31
TOC removal (%)	51.82	57.95	66.82	75.7	83.17	83.06	81.90	82.5	82.57

^a^Organic loading rate

^b^Effluent total suspended solids

^c^Numbers in brackets are methane yield at STP (1 bar and 273.15 °K)

**Table 2:** Performance of up-flow anaerobic fixed film fixed bed reactor (UAnFFFBR) in removing DEP

Study step	A					B			
Study phase	1	2	3	4	5	1	2	3	4
Influent DEP concentration (mg L^−1^)	300	300	300	300	300	400	500	600	700
HRT (h)	12	18	24	30	36	36	36	36	36
*L*_org_ (g COD m^−2^ day^−1^)	4.162	2.775	2.081	1.665	1.387	1.85	2.312	2.775	3.237
Attached mass [TS (mg) of biofilm]	5840	4370	4110	3880	3720	4340	5440	6080	6840
Attached mass [VS (mg) of biofilm]	4080	2940	2700	2460	2280	2880	3660	4320	4980
VS/TS ratio	0.698	0.672	0.657	0.634	0.613	0.663	0.672	0.71	0.728
Effluent total suspended solids (mg day^−1^)	326.8	227.3	169.3	129.6	112.2	144.5	181.4	220	270
Effluent volatile suspended solids (mg day^−1^)	164.4	102.4	74.4	55.7	48	76.8	96	117.6	151.8
DEP concentration in TSS_e_ (mg g^−1^)	9.7	9.3	8.6	8.1	5.2	7.4	7.5	8.4	9.1
SRT (day)	17.87	19.22	24.27	29.94	33.15	30.03	29.98	27.63	25.33
Methane production (L/g COD_rem_)	0.22 (0.2)	0.24 (0.22)	0.28 (0.26)	0.34 (0.31)	0.42 (0.38)	0.34 (0.31)	0.32 (0.29)	0.30 (0.27)	0.27 (0.24)
Methane percentage (%)	41.1	42.3	52.8	56.1	61.5	61.0	59.5	59.6	57.3
DEP removal (%)	67.9	72.5	76.46	80.96	87.86	88.15	88.3	88.36	88.72
COD removal (%)	57.91	60.86	71.85	78.79	83.55	84.25	86.02	86.27	86.91
TOC removal (%)	45.32	53.97	62.58	70.05	78.33	78.34	78.25	78.79	79.65

*L*_*org*_ organic loading rate, *TSS*_*e*_ total solids, *VS* volatile solids, *TSS*_*e*_ effluent concentration of total suspended solids

### Analyzing impacts of hydraulic retention time

In study step (A), the influent concentration of DEP was maintained constant to 300 mg L^−1^ and HRTs were changed from 12 to 36 h. At this stage, the obtained results showed that the DEP removal rate for the AnFFBR can increase from 73.46 to 90.26% with the retention time increase from 12 to 36 h. Pirsaheb et al. [56] found similar results for the suspended growth anaerobic baffled reactor (ABR) and realized that higher HRTs resulted in more COD removal. The related COD and TOC removal efficiencies which showed similar behaviors and maximum removal of COD and TOC was measured as 87.01 and 83.17%, respectively, in 36 h of HRT. The DEP removal in the AnFFBR was considerably higher than its mineralization (COD and TOC removal), which can be related to the presence of benzene ring (aromatic structure) known to be more refractory to biodegradation [8, 57]. The experimental results of study phase (A-1) which compared DEP and TOC removal confirmed this theory. Nearly 21.64% higher removal rate of DEP than TOC showed that the side chains of DEP were biodegraded faster than its benzene ring. This difference reduced to 7.1% by increasing HRT to 36 h.

Similar observations were recorded for the UAnFFFBR. The improved effluent quality of both bioreactors at higher HRTs can be related to the higher contact time of microbial mass to DEP. Another critical factor is solid retention time (SRT), which shows the presence time of microbial mass to fulfill the organic substrate biodegradation. In study step (A), the SRTs of both reactors notably increased by reducing the organic loading rate and increasing HRT. The highest SRTs for study step (A) were observed in 36 h HRT, which were equal to 41.22 and 33.15 (days) for the AnFFBR and UAnFFFBR, respectively. These obtained SRTs were considerably higher than other suspended growth processes (e.g., conventional aerobic activated sludge) ranging from 3 to 30 days. It was also known that SRTs between 5 and 50 days are ideal for xenobiotic compounds to be biodegraded which can be considered as an advantage for both of the studied reactors [30].

The effluent quality of the UAnFFFBR, especially in study phases (A-1) and (A-2), was significantly less than that in the AnFFBR. This result revealed the AnFFBR could tolerate higher organic and hydraulic loading rates than the UAnFFFBR. Greater performance and tolerance of the AnFFBR can be the result of phase separation known as the significant advantage of anaerobic baffled reactors (ABR) [58]. This phenomenon may cause acidogenesis and methanogenesis bacteria to be divided into two different phases in the AnFFBR, which subsequently make it possible for these bacteria to grow and synthesize under their favorable conditions. In this regard, the growth rates of the attached biofilm mass (as mg VS and TS) were higher for the AnFFBR than UAnFFFBR. Furthermore, as the volatile solids (VS)-to-total solids (TS) ratio of the biofilm were higher for the AnFFBR than UAnFFFBR, it can be concluded that the microorganisms had greater activity in the AnFFBR.

Moreover, higher SRTs in the AnFFBR are due to greater persistence of microbial mass to the attained conditions, which consequently leads to more effective bioreaction in the AnFFBR. However, both the bioreactors performed well at 300 mg influent DEP concentration per L and 36 h HRT, resulting in 90.26 and 87.86% of DEP removal to be achieved for the AnFFBR and UAnFFFBR, respectively.

### Analyzing effect of organic loading rate

The bioreactors’ performance under different diethyl phthalate concentrations and organic loading rates (stable hydraulic loading rate of 4 L day^−1^) was evaluated in study step (B). According to the bioreactors’ best performance observed in study phase (B-4), 90.31 and 86.91% of COD and 91.11 and 88.72% of DEP were removed by the AnFFBR and UAnFFFBR, respectively. In this step, the variation of removal rate slightly increased as the substrate and organic loading rate increased from 400 to 700 mg DEP L^−1^ and 1.85 to 3.237 (g COD/m_carrier_^2^ day), respectively. It can be concluded that HRT had a greater impact than organic loading rate on these biofilm reactors. Although the bioreactors’ removal efficiencies increased in study step (B), the COD and DEP concentrations slightly increased in the effluent. Moreover, VS/TS ratio and active biofilm mass are two parameters which can affect the bioreactors’ performance in study step (B). It can be concluded that, by increasing organic loading rate, both the attached microbial masses and their volatile portion increased and, consequently, led to higher biodegradation capacity of the bioreactors (Tables 1, 2). It should be noted that better effluent concentration and performance of the AnFFBR than those of the UAnFFFBR in study step (B) can be related to its higher biofilm mass and VS/TS ratio. Another factor is phase separation which was previously mentioned. Moreover, it has been known that anaerobic reactors’ performance in higher organic loading rates and for strong wastewaters (more than 1000 mg L^−1^ of influent COD) is better compared to when they are used for low strength wastewater treatment [31]. The experimental results of this study (which indicated stable performance of the anaerobic bioreactors) along with Farzadkia et al.’s investigation can demonstrate the advantage of these anaerobic biofilm reactors over aerobic bioreactors [59]. In the study by Farzadkia et al., high organic loading rate of the substrate had an adverse effect on the biodegradation and metabolic activity of aerobic fixed bed activated sludge hybrid reactor, and the removal efficiency of the reactor dropped from about 96–79% when organic loading rate was increased from about 1 to 4.5 kg COD m^−3^ day^−1^ [59].

It should be also noted that the statistical *t* test analyses for DEP, COD, and TOC removal efficiencies showed the mentioned parameters to be statistically different in most of the study phases when compared between the bioreactors (*P* value <0.05).

A summary of some studies conducted on the evaluation of biological removal of PAEs is presented in Table 3. It should be noted that, although all these biological methods indicate that bioremediation can play an important role in removing PAEs, the type and condition of bioreactors including (anaerobic, aerobic, and anoxic conditions), their operational conditions (particularly HRT and SRT), and finally type of the selected phthalate can impact the performance of bioreactors [8, 60–65]. It can be observed that these bioreactors achieve more removal rates as they increase the HRT and SRT, which are in agreement with the observed results of the present study [8, 60, 64]. In addition, there is a diverse relation between the length of alkyl-side chains of phthalates and their biodegradability [8, 61].

**Table 3:** Summary of some studies conducted for biological removal of PAEs

Treatment method	Substrate type (PAEs)	Experimental conditions and removal efficiency	Important observation	Refs.
Anaerobic/anoxic/oxic (AAO) treatment system	DMP	The optimal HRT and SRT for DMP and nutrients removal were 18 h and 15 days, respectively, and the degradation rates of anaerobic, anoxic and aerobic zones for DMP were 13.4, 13.0, and 67.7%, respectively	The biodegradation process of DMP by the selected method was in accordance with the first-order kinetics model. Under the optimal conditions, about 73.8, 5.8, 19.3, and 1.1% of DMP was biodegraded, released in the effluent, accumulated in the system, and remained in the waste sludge, respectively	[60]
Trickling filter	DEP and DEHP	Trickling filter achieved 94–99% of DEP and 44% of DEHP removal	DEHP was the most recalcitrant among the selected phthalates and DEP with less molecular weight was biodegraded with higher rate	[61]
Cyclic activated sludge technology (CAST), anoxic/oxic (AO), and anaerobic/anoxic/oxic (AAO) processes	DEP, DMP, DnBP, BBP, DEHP and DOP	The overall removal efficiency of all the selected PAEs was more than 72% in CAST while AO and AAO only achieved about 30% of PAEs removal	The better performance of the CAST process was attributed to its better indoor-conditions for bacterial community	[62]
Moving bed biofilm reactor (MBBR)	DAP and DEP	In optimum conditions with HRT of 9 h, about 95 and 94% of DEP and DAP were removed, respectively. In addition, more than 92% of COD removal was achieved for both phthalates	MBBR tolerated both of the selected phthalates with the influent concentrations of 100–300 mg L^−1^. Sludge yield constants (*Y*) varied from 0.3 to 0.54. The maximum specific growth rates were 0.37 and 0.34 day^−1^ for DEP and DAP, respectively	[8]
Up-flow anaerobic sludge blanket reactor (UASB)	DMP	More than 99% of DMP and 93% of COD were removed from the wastewater containing 600 mg L^−1^ of DMP (corresponding to 3 g COD L^−1^ day^−1^) of organic loading rate of) at 8 h of HRT	The sludge yield was estimated as 0.08 g VSS g COD^−1^. DMP was first de-esterified to mono-methyl phthalate (MMP) and, then, to phthalate (or phthalic acid) and, subsequently de-aromatized and converted into methane and CO_2_. The maximum specific degradation rates of DMP, MMP, and phthalate were 415, 88, and 36 mg (g VSS day)^−1^, respectively	[63]
Anaerobic/anoxic/oxic treatment system (AAO)	DnBP	The optimal HRT and SRT for DnBP removal were 18 h and 15 days, respectively. In the mentioned conditions, about 72.66% of DnBP was degraded by the selected process, 24.44% was accumulated in the system, 2.44% was released in the effluent, and 0.5% remained in the waste sludge	Increasing SRT from 10 to 15–25 days resulted in increasing removal efficiency from 90 to 95%. Higher SRT improved DnBP biodegradation efficiency. The removal efficiencies of anaerobic, anoxic and oxic reactors were 17.14, 15.02, and 63.46% of the total DnBP removal, respectively	[64]
Submerged membrane bioreactor (aerobic condition)	DEHP	The removal efficiency of DEHP under HRTs of 4 and 6 h, SRT of 140 days, and sludge concentration of 11.5 and 15.8 g VS L^−1^ ranged between 91 and 98%	The removal of DEHP was closely dependent on the membrane pore size and about 74% of inlet DEHP was biodegraded	[65]

*DMP* dimethyl phthalate, *DEHP* bis(2-ethylhexyl) phthalate, *DnBP* di-*n*-butyl phthalate, *BBP* butyl benzyl phthalate, *DOP* di-n-octyl phthalate, *DAP* diallyl phthalate

### Biogas production

Methane, carbon dioxide, hydrogen, and hydrogen sulfide were observed as the main gases in biogas. Both methane production rate and its percentage for bioreactors were affected by HRT and organic loading rate. According to the results, the methane production increased by increasing HRT (study step A) and, then, decreased by increasing organic loading rates (study step B). It is commonly known that operational parameters can critically affect the biogas production rates [66]. The maximum methane production rates were observed in study phase (A-5) with 36 h of HRT as 0.44 and 0.42 L CH_4_ g COD_rem_^−1^ at 1 bar and 298.15 °K (or 0.4 and 0.36 L CH_4_ g COD_rem_^−1^ at 1 bar and 273.15 °K) for the AnFFBR and UAnFFFBR, respectively.

For most of the study phases, the methane produced by the AnFFBR was considerably higher than that produced by the UAnFFFBR, which may be the result of phase separation provided by the baffles of AnFFBR that consequently help the acetogenic bacteria to utilize the volatile fatty acids (VFAs) produced by acidogenic bacteria before these metabolites reach methanogenic bacteria and inhibit their activities. This issue can be confirmed by the results obtained in the study by Wang et al., indicating that some VFAs like propionic acid can significantly inhibit the methanogenesis process [67]. This hypothesis can be supported when the methane yield of the UAnFFFBR in study step B more significantly decreased than that of the AnFFBR by increasing organic loading rate. This phenomenon can be attributed to greater accumulation of some inhibitory compounds (e.g., propionic acid) in the UAnFFBR. Moreover, the study results of step (A) demonstrated that methanogenic bacteria needed more time to convert the VFAs and other metabolites into methane. It can be also observed that increasing HRTs from 12 to 36 h for each of the bioreactors can lead to the increase of the methane production for about twice. These results could be attributed to slow growth and synthesis rate of methanogen bacteria, which results in more SRTs requirement for optimum growth and the substrate concentration increase needed by these bacteria (e.g., acetic acid) in higher HRTs. However, some of the obtained methane yields in the present study, especially in study phase (A-5), were higher for both of the bioreactors compared to some other studies which have commonly reported the methane yields of about 0.27 to 0.36 L CH_4_/g COD [68, 69]. Moreover, the methane production rate is usually expected to be placed around 0.35 L CH_4_/g COD in STP condition (or 0.382 L CH_4_/g COD at 1 bar and 298.15 °K), which is the theoretical methane yield [34, 69, 70]. The obtained higher methane yields in this study might be the result of further conversion of biosolids produced from dead biofilm cells and their lysis into methane and other biogases. It can be confirmed that the effluent volatile suspended solids (VSS_e_) and effluent total suspended solids (TSS_e_)/VSS_e_ ratios were considerably lower for both of the bioreactors in study phase (A-5), as compared to other study phases. As an example, for the AnFFBR, the VSS_e_ and VSS_e_/TSS_e_ ratios in study phase (A-5) were 48.4 mg day^−1^ and 0.537, respectively, which were considerably lower than the VSS_e_ and VSS_e_/TSS_e_ ratios of 224.4 mg day^−1^ and 0.671, respectively, obtained in study phase (A-1). This point illustrates that the remained tissue of dead bacteria was converted into other inert solids and final products including methane.

However, it should be noted that there are many studies which have reported the methane production rates between 0.4 and 0.47 L CH_4_/g COD [71–73].

### Sludge quality

Another essential factor which is particularly important for land application is the concentration of DEP adsorbed by biomass, especially as it is known that the anaerobic digestion of biomass can produce residues that are rich in nutrients and have the potential to be used as the fertilizer [74]. According to the results, DEP can be considerably adsorbed by biofilm and, consequently, observed in effluent TSS. Moreover, the adsorbed concentration of DEP in sloughed biofilm depends on influent DEP concentration and organic loading rate. Minimum DEP concentrations in TSS for both the bioreactors were observed in study phase (A-5) which had the minimum organic loading rate and influent DEP concentration and were as 4.1 and 5.2 mg DEP g TSS^−1^ for the AnFFBR and UAnFFFBR, respectively. It should be noted that, for most of the study phases, the DEP concentrations in TSS were lower in the AnFFBR than UAnFFFBR, demonstrating that the sludge of the AnFFBR can be used more safely. This fact becomes more important when the produced sludge is used for land application and especially for agriculture purposes, because the previous research has shown that some phthalates can reduce the plant growth and cause chloroplast disintegrating [9]; others have stated that they can be introduced into the food chain and, consequently, produce human exposure. However, both the bioreactors’ sludge should further be treated like other sludge. The performed research for evaluating digestion of phthalates in sludge has indicated that such phthalates, particularly short-chains phthalates [e.g., DEP and dimethyl phthalate (DMP)], can be removed and mineralized in significant amounts. However, some other phthalates with long chains like di(2-ethylhexyl) phthalate (DEHP) may show resistance to digestion [6]. Although the produced sludge in the AnFFBR had less adsorbed DEP, the UAnFFFBR had sludge with less VSS/TSS ratio (effluent volatile suspended solids/effluent total suspended solids), which can make it more feasible for digestion and further uses. The minimum and maximum VSS/TSS ratios were observed as 0.537 and 0.671 for the AnFFBR, and 0.427 and 0.562 for the UAnFFFBR [both the minimum ratios were observed in study phase (A-5)]. The minimum VSS/TSS ratio for the UAnFFFBR (0.427) was considerably less than the one reported for the aerobic moving bed biofilm reactor which ranged from about 0.52 to 0.68 [8] and can be considered as an important further sludge management advantage for this anaerobic reactor.

### Metabolic pathways of DEP biodegradation

According to the observed by-products, DEP in both bioreactors was primarily and mainly decomposed to mono-ethyl phthalate (MEP) by the de-alkylation of its first side chain and, then, the bioreaction continued to produce phthalic acid (PA) by removing the other side chain of MEP. This enzymatic reaction is known as de-esterification and commonly reported as the main phthalates’ biodegradation route [75]. In addition, the trace amount of other center metabolites including dimethyl phthalate (DMP) and mono-methyl phthalate (MMP) can be the result of de-methylation which is a less common and predominant biodegradation pathway [75, 76]. Amir et al. [76] reported similar observation and stated that during sludge composting of di(2-ethylhexyl) phthalate (DEHP), DMP can be produced as the by-products of diethyl phthalate biodegradation due to de-methylation pathway. Similar observed metabolites for both bioreactors have indicated that the biodegradation pathway is not different and microbial activity in both bioreactors’ condition can lead to the same by-products.

**Fig. 1:**
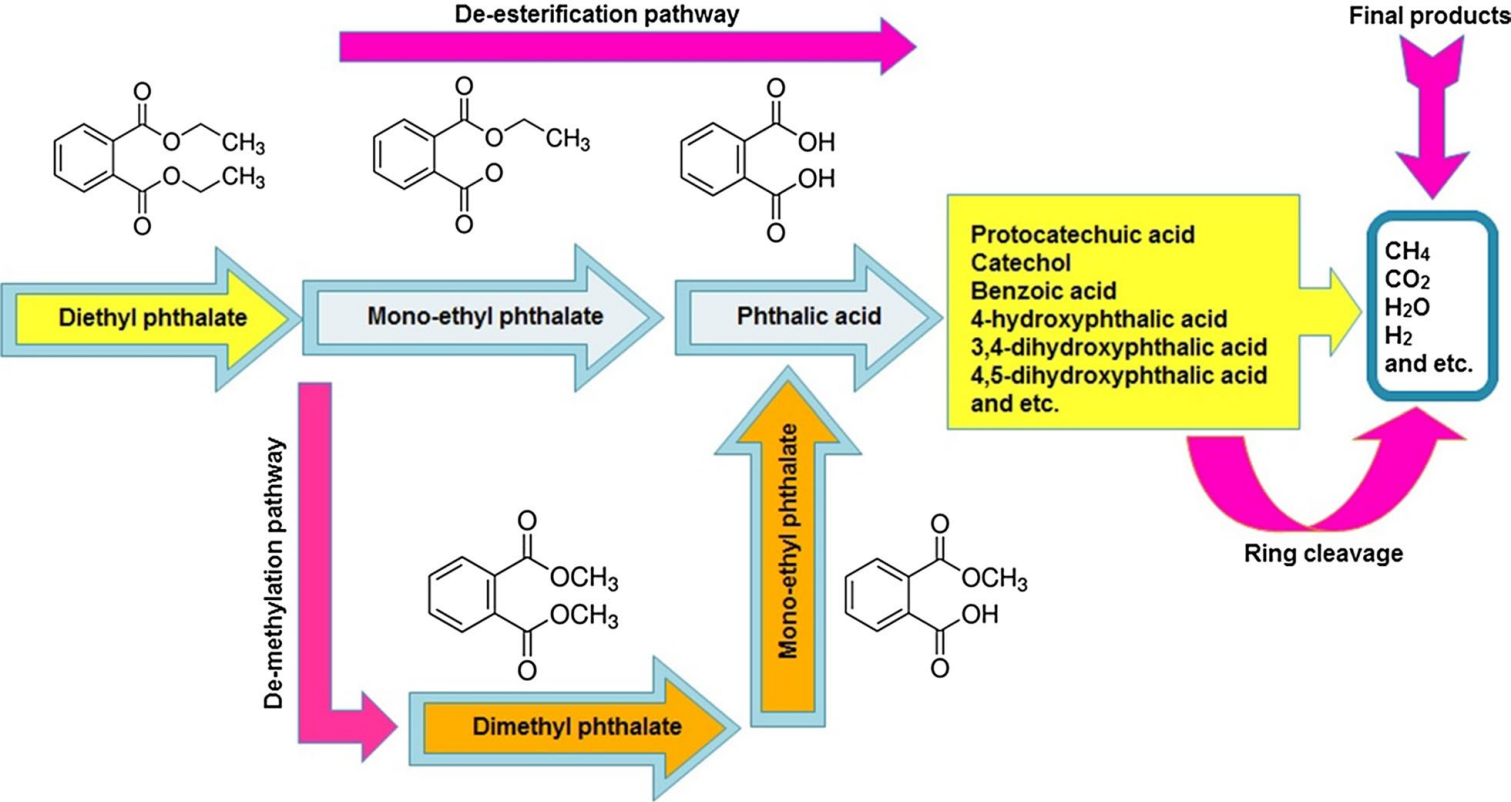
Biological degradation pathway of diethyl phthalate

The other important degradation metabolites produced before ring cleavage included protocatechuic acid, 4-hydroxyphthalic acid, benzoic acid, 4,5-dihydroxyphthalic acid, catechol, and 3,4-dihydroxy benzoic. Then, the biodegradation of the remained benzene ring of the mentioned by-products can result in the production of observed volatile fatty acids (VFAs), which have commonly been reported in the anaerobic biodegradation of organic compounds [77]. The final products of biodegradation were methane, carbon dioxide, dihydrogen oxide, and hydrogen as mentioned before in the biogas. It should be noted that the VFAs and some of the other by-products can be produced in each of the other biodegradation steps. Simplified biodegradation pathway is presented in Fig. 1.

### Modeling and analysis of kinetic coefficients

**Fig. 2:**
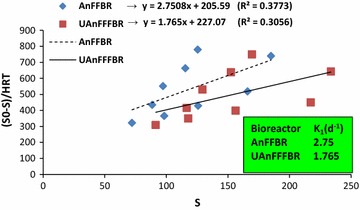
First-order model for the AnFFBR and UAnFFFBR

As shown in Fig. 2, the first-order model for both of the bioreactors had low coefficients of determination, which were 0.377 and 0.305 for the AnFFBR and UAnFFFBR, respectively. Consequently, this model cannot be used for predicting performance of both of the bioreactors.

**Fig. 3:**
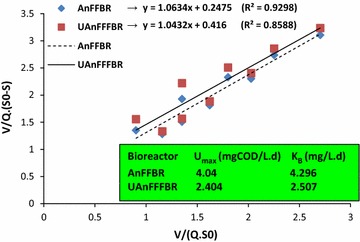
Stover–Kincannon model for the AnFFBR and UAnFFFBR

For computing saturation value constant (*K*_B_) and maximum substrate removal rate (*U*_max_), Eq. (4) was plotted for both bioreactors in Fig. 3. Moreover, semi-strong values of coefficient of determination were obtained as 0.929 and 0.858 for the AnFFBR and UAnFFFBR, respectively, indicating that this model can be applied for designing and predicting the selected bioreactors. Higher *U*_max_ and *K*_B_ of 4.04 (mg COD L^−1^ day^−1^) and 4.296 (mg L^−1^ day^−1^) for the AnFFBR compared to 2.404 (mg COD L^−1^ day^−1^) and 2.507 (mg L^−1^ day^−1^) for the UAnFFFBR, respectively, demonstrated that the microbial community had better conditions for biodegrading DEP and stabilizing COD in the AnFFBR than UAnFFFBR.

**Fig. 4:**
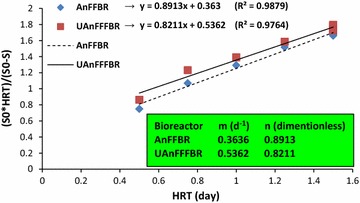
Second-order (Grau) model for the AnFFBR and UAnFFFBR

Equation (6) was plotted in Fig. 4 for computing *K*_G_ coefficient. After calculating (*m*) and (*n*) coefficients, *K*_G_ was determined using Eq. (7). Table 4 presents the Grau coefficient (*K*_G_) values. This model clearly had a high degree of precision with the coefficients of determination of 0.987 and 0.976 for the AnFFBR and UAnFFFBR, respectively. In this regard, the Grau model can be used for predicting the performance of both of the bioreactors. Evaluating the *K*_G_ values demonstrated this model to be in compliance with the obtained results, including COD, DEP, and TOC removal, because the *K*_G_ values for the AnFFBR were higher than those for the UAnFFFBR. In study step (A), as HRT increased, the *K*_G_ values in the reactors were significantly raised, which may be due to the bioavailability of microorganisms to the substrate. In study step (B), the *K*_G_ values did not considerably change. With regard to the increasing DEP concentration and organic loading rate in study step (B) and stability of the calculated *K*_G_, it can be concluded that both of the bioreactors can tolerate high organic loading rate and their performance was not affected by DEP concentration. This point can be the advantage of these anaerobic systems over aerobic or other treatment processes for industrial wastewater treatment [30].

**Table 4:** Grau second-order kinetic coefficients (*K*_G_) for AnFFBR and UAnFFFBR

Study phase	A-1	A-2	A-3	A-4	A-5	B-1	B-2	B-3	B-4
*K*_G_ for AnFFBR	2.038	2.779	3.253	3.64	3.822	3.92	3.92	4.023	4.01
*K*_G_ for UAnFFFBR	1.522	2.112	2.300	2.524	2.723	2.875	2.828	2.875	2.909

According to Table 5 [correlation of methane yields (L CH_4_/g COD_rem_) and Grau coefficients], although in study step (B) for both bioreactors, strong correlation (*R* ^2^ > 0.7) between methane production rates and *K*_G_ values was not observed and, therefore, *K*_G_ values could not be used for predicting methane yield with a high degree of precision, strong coefficients of determination (*R* ^2^) of study step (A) as 0.791 and 0.937 for the UAnFFFBR and AnFFBR, respectively, can be used for the mentioned prediction. Positive slopes of the obtained equations in study step (A) (0.156 and 0.117 for the UAnFFFBR and AnFFBR, respectively) can demonstrate that HRT had a positive impact on both *K*_G_ and methane yield values and these dependent variables changed with a similar direction. Higher calculated slope for the UAnFFFBR than AnFFBR can demonstrate that HRT had a more impact on methane production of the UAnFFFBR and, considering lower obtained methane yields for the UAnFFFBR, the AnFFBR had more advantages due to its less dependence on HRT. Furthermore, with regard to the insignificant variations of the calculated *K*_G_ and negative slopes of study step (B) as 0.56 and 0.39 for the UAnFFFBR and AnFFBR, respectively, it can be concluded that methanogens activities were affected more than other non-methanogens by higher DEP loading rates. In addition, higher negative slope for the UAnFFFBR can demonstrate that this bioreactor was more sensitive to higher organic and DEP loading rates and its methane yield was more affected.

**Table 5:** Correlation of methane yields (L CH_4_/g COD_rem_) and Grau coefficients

	UAnFFFBR	AnFFBR
Study phase A	*Y* = 0.117*x* − 0.041 (*R* ^2^ = 0.937)	*Y* = 0.156*x* − 0.05 (*R* ^2^ = 0.791)
Study phase B	*Y* = −0.39*x* + 1.892 (*R* ^2^ = 0.571)	*Y* = −0.560*x* + 1.917 (*R* ^2^ = 0.390)

**Fig. 5:**
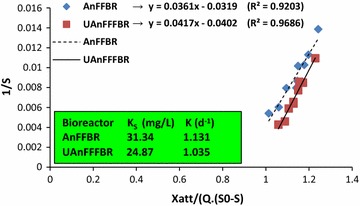
Mathematical calculation of (*K*_S_) and (*K*) for the AnFFBR and UAnFFFBR

The kinetic coefficients including (*K*_S_) and (*K*) were computed using Eq. (10), which is plotted in Fig. 5. Half saturation constant and overall reaction rate were computed as 31.34 mg L^−1^ and 1.13 day^−1^ for the AnFFBR, and 24.87 mg L^−1^ and 1.03 day^−1^ for the UAnFFBR, respectively. It should be noted that the anaerobic wastewater treatment has slower substrate utilization rate as one of their disadvantages [31], which can be confirmed by the obtained overall reaction rates. As an example, for an aerated submerged fixed film reactor with glucose as the substrate, the overall reaction rate has been computed about 2.7 day^−1^, while for other conventional aerobic treatments, it is reported up to 12 day^−1^ [30, 49]. Half saturation constant presents the substrate concentration at half of the maximum substrate utilization rate. The previous studies have declared that a substrate with higher biodegradability has more *K*_S_ [8]. Under low substrate availabilities and at the same maximum specific growth rate in the two microbial groups or even two different bioreactors, smaller *K*_S_ is obtained for microbial mass or a bioreactor with higher affinity to the substrate [78]. As the calculated maximum growth rate (*µ*_m_) for the selected bioreactors is different, *K*_S_ coefficients should not be directly applied to compare the bioreactors’ performances. By contrast, if the difference between two *µ*_m_ values is ignored, the UAnFFFBR can be stated to have better performance under low substrate concentrations and, therefore, the microbial growth will be less influenced by substrate concentration. It is a very important point of view, especially for industries having different work shifts, as it does not continuously generate wastewater or the substrate concentration is not stable in influent wastewater.

**Fig. 6:**
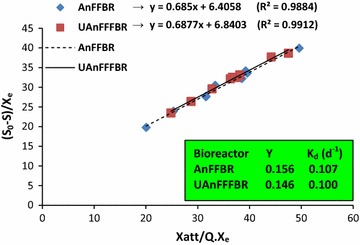
Mathematical calculation of (*Y*) and (*K*_d_) for the AnFFBR and UAnFFFBR

The computed biomass yield coefficients (*Y*) plotted in Fig. 6 were obtained as 0.156 and 0.146 (g VS produced/g COD utilized). The (*Y*) coefficient is an important parameter for sludge management and its subsequent disposal [77, 79]. From this point of view and without other removal performances, the UAnFFFBR can be preferably used when strong wastewater with high hydraulic loading rate must be treated. Under this circumstance, the insignificant difference observed for (*Y*) between the selected bioreactors results in sludge production to differ significantly. Meanwhile, the biomass yields for both bioreactors are very desirable when compared to those for aerobic based reactors. Experimental results for the biodegradation of similar compounds in aerobic bioreactors have shown that they have biomass yields more than 0.5 or even up to 0.78, which is more than five times greater than the calculated (*Y*) in this study [8, 48]. The *K*_d_ coefficients, which present specific decay rate (and are expressed as g VS of microbial mass loses/g VS of presents mass day or day^−1^) were computed as 0.107 and 0.100 day^−1^ for the AnFFBR and UAnFFFBR, respectively. The difference of the values, however, was observed to be insignificant between the bioreactors. These values placed in the typical range of 0.06–0.15 day^−1^ for the conventional treatment [30].

Consequently, maximum specific growth rate (day^−1^) values were calculated from Eq. (15) as 0.176 and 0.151 day^−1^ for the AnFFBR and UAnFFFBR, respectively. This coefficient had good compliance with other experimental results (including COD removal) and with the Stover–Kincannon and second-order (Grau) models, indicating that the AnFFBR had better performance. Considering the same inoculation used for both of the bioreactors, *µ*_m_ demonstrated that the microbial mass had more favorable conditions in the AnFFBR. Finally, (*r*_su_) coefficients were computed for the study phases with maximum COD removal (phase B-4 for both of the bioreactors). Higher *r*_su_ of 938.11 mg L^−1^ day^−1^ for the AnFFBR compared to 842.26 mg L^−1^ day^−1^ for the UAnFFFBR confirmed other experimental results and proved the AnFFBR to have better performance due to its faster utilization rate.

## Conclusions

Both the bioreactors were capable of treating low-to-high strength DEP wastewater; however, the AnFFBR was preferred, since it could achieve 90.31% COD removal at 36 h of HRT. This study suggested the Grau and Stover-Kincannon models for predicting bioreactors due to their suitable coefficients of determination and good conformity of their kinetic parameters to the obtained results. Moreover, the experimental results and obtained kinetic coefficients indicated that the AnFFBR had better performance than the UAnFFFBR. Although both these bioreactors can achieve nearly 90% of DEP removal, they are promising only as pre-treatment methods and, due to standard regulations, should be coupled with further technology. Although from the sludge management standpoint, the AnFFBR can be considered as a safer treatment option than the UAnFFFBR due to its lower DEP concentrations in sludge, the UAnFFFBR had less VSS/TSS ratio, which makes it more practical for digestion. Moreover, the AnFFBR had more sludge yield which should also be taken into consideration. Finally, both the bioreactors showed considerable methane yield; however, compared to the UAnFFFBR, the AnFFBR had more potential for bioenergy production, which could result in saving more energy and costs.

## Additional file

**Additional file 1: Figure S1.** Schematic diagram of the (A) UAnFFFBR; and (B) AnFFBR.

## Abbreviations

DEP: diethyl phthalate
AnFFBR: anaerobic fixed film baffled reactor
UAnFFFBR: and up-flow anaerobic fixed film fixed bed reactor
CNEMC: China National Environmental Monitoring Center
USEPA: United State Environment Protection Agency
COD: chemical oxygen demand
MWTP: municipal wastewater treatment plant
HDPE: high-density polyethylene
HRT: hydraulic retention time
GC: gas chromatograph
FID: flame ionization detector
MS: mass spectrometer
TOC: total organic carbon
VS: volatile solids
TS: total solids
VSS: volatile suspended solids
TSS: total suspended solids
MEP: mono-ethyl phthalate
PA: phthalic acid
MMP: mono-methyl phthalate
DEHP: di(2-ethylhexyl) phthalate
DMP: dimethyl phthalate
VFAs: volatile fatty acids
STP: standard temperature and pressure
L_org_: organic loading rate
SRT: solid retention time
