# Supplementary figures and images for: A comparative study of anaerobic fixed film baffled reactor and up-flow anaerobic fixed film fixed bed reactor for biological removal of diethyl phthalate from wastewater: a performance, kinetic, biogas, and metabolic pathway study
Source: Biotechnol Biofuels. 2017 May 31;10:139. doi: 10.1186/s13068-017-0826-9 (PMC5452402; doi:10.1186/s13068-017-0826-9)

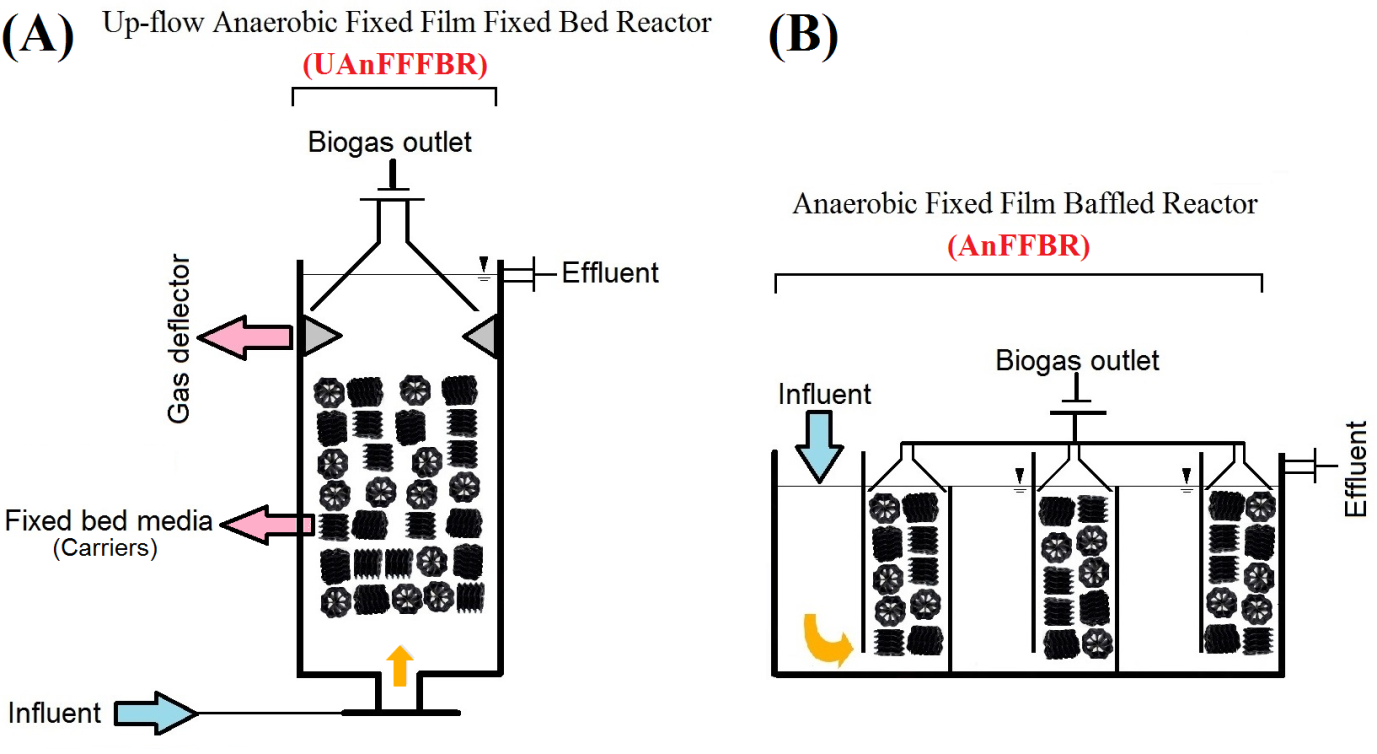


Figure S1: Schematic diagram of the (A) UAnFFFBR; and (B) AnFFBR.

Supplement: Supplementary file 1 — Additional file 1: Figure S1. Schematic diagram of the (A) UAnFFFBR; and (B) AnFFBR. [file 13068_2017_826_MOESM1_ESM.docx]
